# First detection of New Delhi metallo-β-lactamases variants (NDM-1, NDM-2) among *Pseudomonas aeruginosa* isolated from Iraqi hospitals

**Published:** 2018-04

**Authors:** Sarah Jehad Ismail, Suhad Saad Mahmoud

**Affiliations:** Department of Biotechnology, University of Baghdad, AL Mansour City, Baghdad, Iraq

**Keywords:** *P. aeruginosa*, Carbapenem resistance, *bla*_NDM_ variants 1, 2

## Abstract

**Background and Objectives:**

Multidrug resistance and in particular, carbapenem resistant Gram-negative bacteria is spreading worldwide at an alarming rate. Among the clinically significant carbapenemases, the New Delhi Metallo-β-lactamase (NDM) is one of the most formidable. NDM efficiently hydrolyses β-lactams and is the last-resort among carbapenems. Hence, therapeutic options for NDM producer bacteria become restricted to a handful of antibiotics. The present study was undertaken to detect the prevalence of the *bla*_NDM_-variants Metallo β-lactamases (MBLs) among isolates of *Pseudomonas aeruginosa* recovered from various clinical samples of hospitalized patients in Baghdad, Iraq.

**Materials and Methods:**

A total of 100 isolates of Gram-negative bacteria obtained from various clinical samples were subjected to antibiotic susceptibility testing by the disc-diffusion method against meropenem (10 μg), imipenem (10 μg), doripenem (10 μg), polymyxin B (10 μg), colistin (10 μg), amikacin (30 μg), gentamicin (10 μg), aztreonam (30 μg), ciprofloxacin (5 μg), levofloxacin (5 μg), ofloxacin (5 μg), cefepime (30 μg), ceftazidime (30 μg), piperacillin-tazobactam (100\10 μg), tigecycline (15 μg) and tetracycline (10 μg). The results were interpreted according to the guidelines suggested by the Clinical Laboratory Standards Institute. Presence of *bla*_NDM_ was detected by PCR and it was confirmed by DNA sequencing of the gene present in the isolates that exhibited carbapenem resistance.

**Results:**

In the present study, four isolates of *P. aeruginosa* carried the *bla*_NDM_, three isolates harboured *bla*_NDM-1_ and one isolate harboured *bla*_NDM-2_. All isolates were resistant to imipenem and meropenem. The *bla*_NDM-1_ carrying isolates remained susceptible to colistin and β-lactamase inhibitors piperacillin-tazobactam.

**Conclusion:**

We are reporting emergence of the *P. aeruginosa* carrying the *bla*_NDM_-variant, which exhibited resistance to imipenem and meropenem for the first time in Iraq.

## INTRODUCTION

Carbapenems are amongst the most effective drugs and first line treatment for infections caused by extended-spectrum β lactamase (ESBL) and AmpC β-lactamase-producing Gram-negative bacteria, including *Enterobacteracea, Pseudomonas* and *Acinetobacter* species.

Carbapenemase is classified according to the Ambler categorization system into A, B and D carbapenemases, are subdivided into different classes of β-lactamases based upon the hydrolytic mechanisms in their active sites. Serine carbapenemases are included in classes A and D, which are referred to as being serine dependent because they have serine (amino acid) in the active site, whereas class B carbapenemases have zinc (zinc dependent) and are referred to as metallo-β-lactamases. Ambler class-A carbapenemases are inhibited by clavulanic acid (a β-lactamase inhibitor) and can be encoded by plasmid and chromosomes. The most common enzymes in this class are *Serratia marcescens* enzyme (SME), Non-metallocarbapenemase-A (NMC), and *Klebsiella pneumoniae* carbapenmase (KPC), which are frequently found in *Klebsiella pneumoniae*. Class B metallo-β-lactamases are plasmid-encoded (in some cases chromosomal) and the most common enzymes include the Verona integron–encoded metallo-β-lactamases (VIM), Impemase (IMP), Sao Paulo MBL (SPM), German imipenemase (GIM), Seoul Imipenemase (SIM) and the New Delhi Metallo-β-lactamase (NDM) families (1, 2).

Amongst the clinically significant carbapenemase enzymes, the New Delhi metallo-β-lactamase (NDM) is currently considered a major concern due to its rapid spread worldwide. This enzyme is efficient in hydrolysing β-lactams and last-resort carbapenems; it is predominantly associated with *Enterobacteriaceae* and *Acinetobacter* spp. but has been much less frequently detected in *Pseudomonas aeruginosa.*

Before 2012, only one report of two NDM-1-producing *P. aeruginosa* isolates was described and both were isolated in Serbia (3), and since then, it has been isolated in different parts of Europe, Egypt and India (4). In Iraq, the first report about the dissemination of NDM-producing *K. pneumoniae* was reported from France in 2010 from an Iraqi trauma patient referred to France for treatment (5). Subsequently, in 2014, Alsharaetal published the first article that documented presence of NDM-1-producing *Pseudomonas* isolates in Najaf, Iraq (6). To the best of our knowledge, these are the only two studies, prior to this study, that have documented the presence of NDM variants 1 and 2-producing *P. aeruginosa*.

## MATERIALS AND METHODS

### Strain identification and drug sensitivity tests.

For the purposes of this study, a total of 100 isolates were collected from wounds, respiratory tract, urine and ear swabs of patients admitted to different hospitals in Baghdad. The API 20 NE biochemical identification system for Gram-negative bacteria was used to identify bacteria, using the methods described by the manufacturer (bioMérieux, France).

All isolates were subjected to antibiotic susceptibility tests that were performed on Mueller Hinton agar plates using the Kirby-Bauer disc diffusion method, and the results were interpreted according to the guidelines suggested by the Clinical Laboratory Standards Institute (7). The antibiotics used were meropenem (10 μg), imipenem (10 μg), doripenem (10 μg), polymyxin B (10 μg), colistin (10 μg), amikacin (30 μg), gentamicin (10 μg), aztreonam (30 μg), ciprofloxacin (5 μg), levofloxacin (5 μg), ofloxacin (5 μg), cefepime (30 μg), ceftazidime (30 μg), piperacillin-tazobactam (100\10 μg), tigecycline (15 μg) and tetracycline (10 μg).

**Phenotypic detection of carbapenemase: Carbapenemase confirmatory test:** The Carba-NP Confirmatory test was used in order to identify carbapenemase production in *Enterobacteriaceae, Pseudomonas* and *Acinetobacter* spp. The test was performed as follows (8): two microcentrifuge tubes were labelled A and B. For each isolate to be tested, a 1-μL loopful of bacteria from an overnight blood agar plate was emulsified in both tubes. Then, 100 μl from a protein extract solution in Tris-HCL (pH 7.4) was added to each tube. After that, 100 μl of Solution A (10 mM Zinc sulphate heptahydrate solution, 0.5% phenol red solution, 0.1 N sodium hydroxide solution) was then added to tube A, and 100 μl of Solution B (Solution A + 6 mg\ml Imipenem) powder was added to tube B. Both tubes were then incubated at 37°C for up to 2 hours. A positive result indicator for Tube A would be a red, or red-orange solution, while a positive result indicator for Tube B would be an orange, yellow, or dark yellow solution.

### EDTA combined disc test (EDTA-CDT).

This test was performed according to the method suggested by Galani et al. (9) as follows: Two 10 μg MEM discs and two 10 μg IPM discs were placed on a plate inoculated with the test organism, and 10 μl of 0.5M EDTA solution was added to one disc of MEM or IPM. The inhibition zones of the MEM and MEM+EDTA, or IPM and IPM+EDTA discs were compared after overnight incubation. A difference in zone diameter between any of the discs alone and with EDTA ≥7 mm was interpreted as a positive test result.

### Molecular detection of *bla*_NDM_.

The extraction of DNA was carried out using two different methods. The first method was colony PCR performed as follows (10): the fresh bacterial culture was put into 100–200 μl of sterile deionized water, boiled for 10 minutes and then subjected to centrifugation at 14,800 × g for five minutes. The supernatant was extracted and preserved at −20°C to use as DNA template. The second method involved extracting the chromosomal DNA from carbapenemase-producing isolates using the G-Spain extraction kit (South Korea) according to the manufacturer’s instructions.

The detection of the *bla*_NDM_-variant was carried out using the PCR molecular technique. The primers assessed in this study were NDM: FW: ATGGAATTGCCCAATATTATGC and NDM: RE: CGAAAGTCAGGCTGTGTTG which were amplified in fragment sizes of 490 bp (11).

Amplification was performed under the following thermal cycling conditions: 10 minutes at 95°C; 35 cycles of amplification consisting of one minute at 95°C, 40 seconds at 57°C, 50 seconds at 72°C and then a further 10 minutes at 72°C for the final extension. DNA fragments were visualized by electrophoresis in a 2% agarose gel at 100V for one hour in 1 TAE (40 mMTris-HCl pH (8.3), 2 mM acetate, 1 mM EDTA) containing 0.05 mg/L ethidium bromide. Using this technique, the *bla*_NDM-1_-positive isolates were detected in less than three hours with 100% sensitivity and excellent specificity. The amplicons were then submitted to the Macron Company (South Korea) for DNA sequencing. Finally, sequence alignment was performed by using Basic Logic Alignment Search Tools (BLAST) available at the National Centre for Biotechnology Information (NCBI).

## RESULTS

### Screening for metallo-enzyme (MBL) production.

**Fig. 1:**
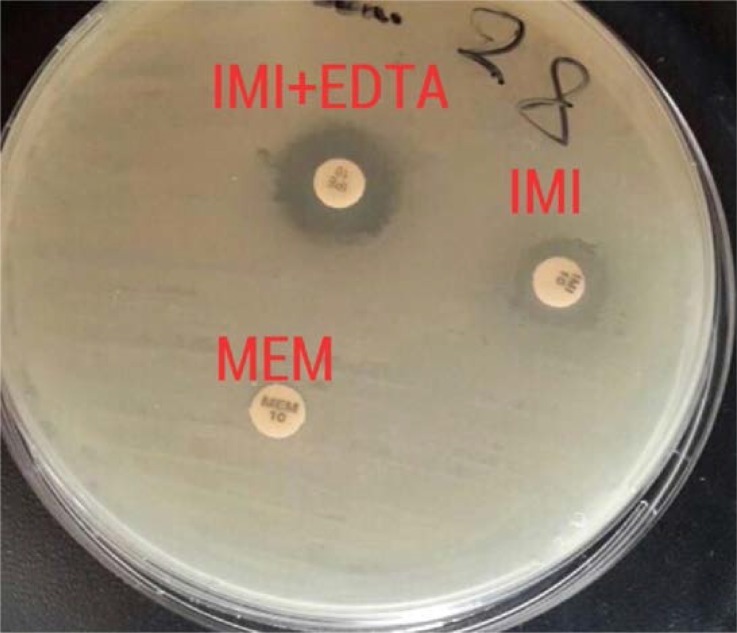
Combined disk for detection of metallo-β-lactamase. β-lactamase producer strain shows a synergistic zone of inhibition between imipenem and imipenem EDTA disc.

Altogether, 100 different isolates, 22 of which were isolates of *P. aeruginosa* were screened for carbapenemase (MBL) production. Out of 22 isolates, six (No. 28, 38, 42, 76, 90 and 93) were carbapenem resistant and were tested positive for metallo-β-lactamase production according to the phenotypic test (EDTA-CDT and CarbNP) Fig. 1.

**Fig. 2:**
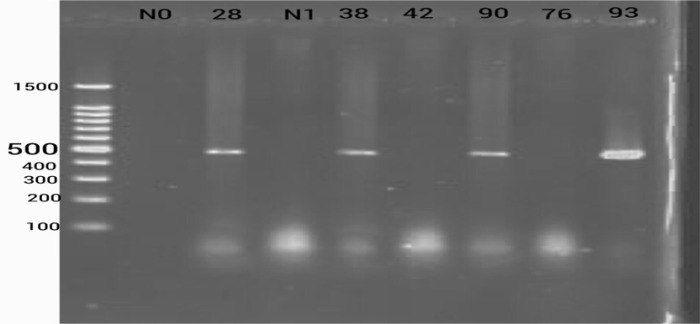
Agarose gel electrophoresis (2%) represents 490 bp *bla*_NDM-1,2_ in *P. aeruginosa*., lane (1) ladder (1500pb), 2) N0 (empty well), 3) *P. aeruginosa* 28 (NDM1 positive), 4) N1, (negative control), 5) *P. aeruginosa* 38 (NDM1 positive), 6) *P. aeruginosa* 42 (NDM1 negative), 7) *P. aeruginosa* 90 (NDM1 positive), 8) *P. aeruginosa* 93 (NDM2 positive).

The six carbapenem resistant isolates were screened by PCR with *bla*_NDM-1_ specific primers. Of these, four isolates were positive for *bla*_NDM_ and yielded 490 bp (Fig. 2). Amplification results were validated by sequencing and the sequencing of *bla*_NDM_ from three isolates (28, 38, 90) were from pneumonia, UTIs and foot ulcers showed identity with *bla*_NDM-1_ (97, 98 and 100% respectively) (Gene bank KU510370.1) while isolate 93, which was isolated from a diabetic patient with an amputated foot showed 98% identity with *bla*_NDM-2_ (Gene bank KU510393.1). The *bla*_NDM-1_ and 2 sequences obtained in this study has been deposited into Genbank under the accession number MF417626.1 and MF542295.1

All data related to the microbial cultures, phenotypic detections and genetic material sources are shown in Table 1.

**Table 1:** Phenotypic and molecular characterization for NDM-positive isolates

**Isolate No.**	**Isolation Source**	**Carba – NP TEST**	**imipenem 10 μg**	**imipenem 10 μg-MBL inhibitor**	***bla*_NDM_**	**Gene location**
28	Pharyngeal swab	Positive	9	14	Positive	Plasmid
38	Urine	Positive	10	17	Positive	Chromosomal
76	Wound	Positive	0	20	Negative	-
42	Bronchial	Positive	10	15	Negative	-
90	Diabetic ulcer	Positive	11	19	Positive	Chromosomal
93	Diabetic ulcer	Positive	11	19	Positive	Chromosomal

All carbapenem non-susceptible isolates were found to be MDR-positive except for isolate number 28 which was a pan drug. The results revealed that isolates were resistant to β-lactam, tetracyclines, cephalosporins and fluoroquinolones. In contrast, these isolates were sensitive to aminoglycosides, monobactam and lipopeptides except for isolate number 28 which was found to be resistant.

**Fig. 3:**
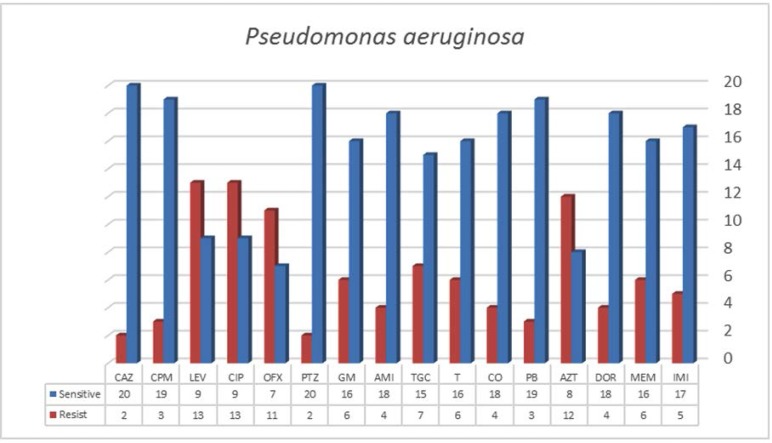
Antibiotic susceptibility of *bla*_NDM_ gene-positive and negative *Pseudomonas aeruginosa* Abbreviations; IMI:Imipenem, MEM: Meropenem, DOR: Doripenem, PB: Polymyxin, CO: Colistin, T: Tetracycline, TGC: Tigecycline, AZT: Aztreonam, AMI: Amikacin, GM: Gentamycin, PTZ: Piperacillin-Tazobactam, OFX: Ofloxacin, CIP: Ciprofloxacin, LEV: Levofloxacin: CPM:cefepime, CAZ: Ceftazidime.

Regarding carbapenem-susceptible *P. aeruginosa* isolates, the results of the antibiotic susceptibility testing revealed high rates of sensitivity to the cephalosporins group including cefepime (86.4%) and ceftazidime (90.9%), piperacillin-tazobactam (90.9%), followed by colistin (81.8%) polymyxin (86.4%), amikacin (81.8%) gentamicin (72.2%). Their sensitivity to imipenem, meropenem and doripenem were 73.9%, 68.2% and 81.8% respectively. Additionally, tetracycline antibiotics showed more sensitivity (72.7%) than tigecycline (68.2%), whereas these isolates were shown to be resistant to fluoroquinolones including: ciprofloxacin, levofloxacin (59. 1%), ofloxacin in (68.2%) and aztreonam (63.6%) (Fig. 3).

## DISCUSSION

In this study, we were the first team to report the presence of the *bla*_NDM_-variant among *P. aeruginosa* isolates. Several phenotypic confirmation tests have been reported to detect carbapenemase production among Gram-negative bacteria. In our study, the EDTA-CD test was used and our results revealed that this test was a reliable and effective method to detect the production of MBL by carbapenem non-susceptible isolates. The results achieved are consistent with Nordman et al. (12) who successfully detected MBL production achieved by the EDTA inhibition test using the double disc test. The main limitation of this assay is that investigators may fail to detect carbapenem non-susceptible isolates that have low level resistance. Moreover, EDTA-containing discs are often prepared manually which increases the chances of errors.

Regarding the antibiotic susceptibility testing results, all carbapenem non-susceptible isolates were resistant to most antibiotics except aminoglycosides and colistin, while one isolate was shown to be resistant to the aminoglycoside group. Our results are inconsistent with Yong et al. and Berger (13, 14) who documented that most NDM producers are typically aminoglycoside resistant because they always carry a 16S rRNA methylase gene (*rmtC* and *armA*) together with the NDM gene on mobile elements.

In addition, susceptibility profiling indicates lower resistance rates to colistin antibiotic while these isolates showed a high level of resistance to tigecycline antibiotic. Our results were consistent with the findings of Rahman et al. (15) (except for tigecycline) who found that NDM-1 producers always appeared susceptible to these particular antibiotics. The results also showed high resistance to the antibiotic ceftazi-dime. Moreover, Quinolone resistance may have resulted from chromosomal mutations in the Quino-lone-resistance determining region in DNA gyrase or by the plasmid-mediated Quinolone resistance determinant *qnrB*. It is likely that the high levels of quinolone resistance seen in our isolates is mediated via this mechanism. Although aztreonam is stable to hydrolysis by MBL producers, our results showed moderate level of aztreonam resistance.

From sequence analysis, two variants of *bla*_NDM-1_ and 2 were detected and according to our research we believe this is the first detection of NDM2 worldwide. Many studies mentioned that NDM-2 resulted from substitutions of Proline by Alanine at position 28, according to results of comparative analysis of NDM-1 and NDM-2 made by Kaase et al. (16) through cloning experiments showing that there are relative contributions to carbapenem resistance and they share the same spectrum of hydrolysis. However, to date, the transfer mechanisms, location, and *bla*_NDM_ genetic environment in *P. aeruginosa* remain unknown. It is important to note that carbapenemase enzymes are not the only mechanisms of acquired resistance to carbapenems. There are also other resistance mechanisms in *P. aeruginosa* including upregulated efflux pumps and the loss of the outer membrane protein encoding gene *oprD* (17) .

In conclusion, to the best of our knowledge, this is the first study in which the *bla*_NDM_ variant in *P. aeruginosa* has been detected in Baghdad hospitals. The fact that this extremely drug-resistant isolate can be transmitted highlights the gravity of this escalating public health issue.
